# Pulmonary thromboembolism—a rare presentation for Strongyloides hyperinfection in an immunocompetent patient

**DOI:** 10.1093/omcr/omae152

**Published:** 2024-12-10

**Authors:** Mir Wasim Ali, Soumadip Rakshit, Atreyee Sarkar, Md Karimulla Mondal, Anup Kumar Datta, Uttara Chatterjee

**Affiliations:** Department of Internal Medicine, IPGMER & SSKM Hospital, 242 Harish Mukherjee Road, Kolkata 700020, India; Department of Internal Medicine, IPGMER & SSKM Hospital, 242 Harish Mukherjee Road, Kolkata 700020, India; Department of Pathology, IPGMER & SSKM Hospital, 242 Harish Mukherjee Road, Kolkata 700020, India; Department of Internal Medicine, IPGMER & SSKM Hospital, 242 Harish Mukherjee Road, Kolkata 700020, India; Department of Internal Medicine, IPGMER & SSKM Hospital, 242 Harish Mukherjee Road, Kolkata 700020, India; Department of Pathology, IPGMER & SSKM Hospital, 242 Harish Mukherjee Road, Kolkata 700020, India

**Keywords:** autoinfection, bronchoalveolar lavage, larva, immunocompetent, nematode, strongyloidiasis, tropical, thromboembolism

## Abstract

*Strongyloides stercoralis* is an intestinal nematode. It is widely distributed in the tropics and sub-tropics of the world. It can cause a wide array of illnesses ranging from asymptomatic autoinfection to a severe form of hyperinfection and disseminated strongyloidiasis. Here we report a case of a 73-year-old patient presenting with obstructive shock with bipedal edema without any evidence of adrenal insufficiency. An upper gastrointestinal endoscopy revealed *Strongyloides* larva in the lumen of duodenal crypts. Stool examination and broncho-alveolar lavage revealed the presence of S. stercoralis larva. Computed tomography of pulmonary angiography revealed pulmonary thromboembolism. A long segment thrombus was also noted in the right external iliac vein on color Doppler with negative screening for malignancy on computed tomography scan. This case suggests that pulmonary thromboembolism with anemia and hypoalbuminemia could be a rare presentation of a systemic illness like Strongyloides hyperinfection in an immunocompetent patient from the tropics.

## Introduction

Strongyloidiasis is an infection that is caused by the soil-transmitted female nematode *Strongyloides stercoralis* that inhabits in small intestines of humans. This nematode is predominantly found in tropical and subtropical areas of the globe and is thought to infect 30 to 100 million people worldwide [1]. It is a unique helminth that inherits the property of autoinfection and proliferation in the host for an indefinite time, remaining in a dormant phase and causing mild eosinophilia in peripheral blood smear or increased serum IgE [2]. More than 50% of chronically infected patients remain asymptomatic until there is a decrease in immunity in the host that potentiates this organism to cause disseminated strongyloidiasis with deposition of the nematode in various tissues of the human body, activating the proinflammatory and prothrombotic states and sometimes leading to thromboembolic events [3]. Here, we present a rare case of *Strongyloides* hyperinfection in an immunocompetent host that presents as pulmonary thromboembolism.

**Figure 1:**
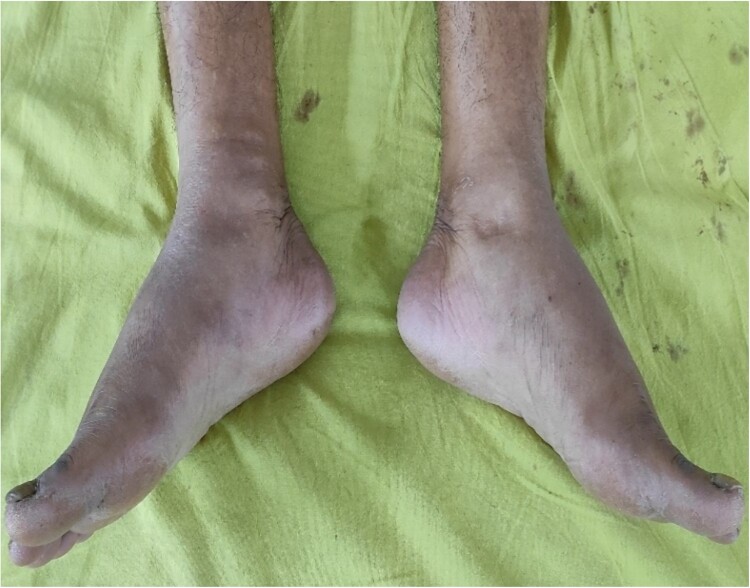
Bipedal pitting edema.

**Figure 2:**
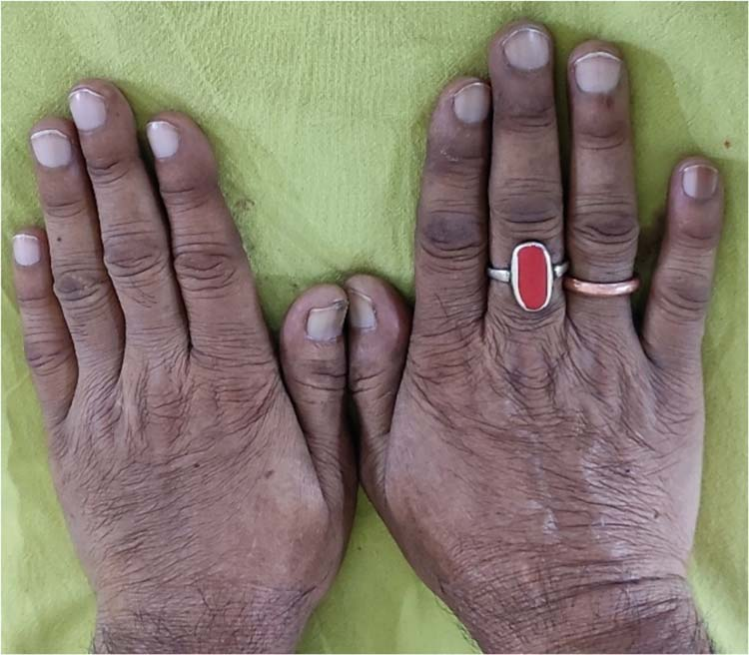
Leukonychia.

## Case report

**Figure 3:**
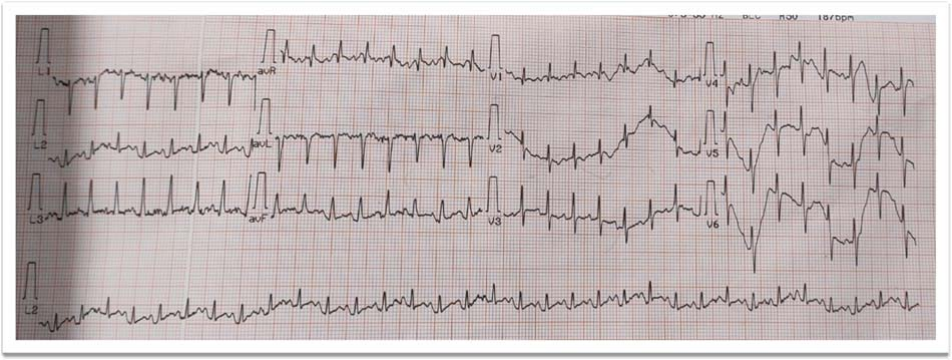
Electrocardiogram showing sinus tachycardia.

**Figure 4:**
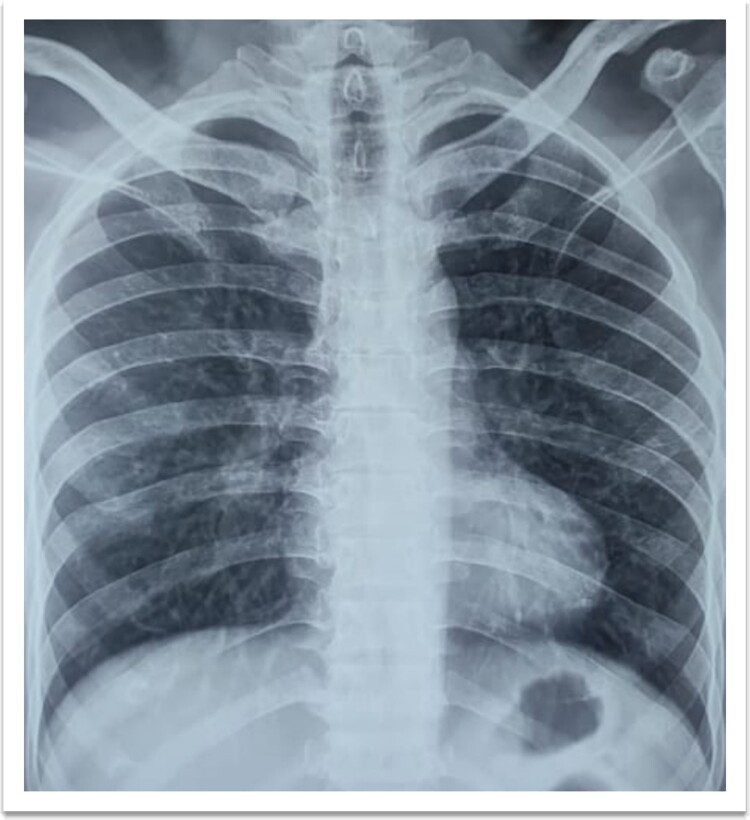
Chest X-ray PA view—No evidence of pulmonary infiltrates.

**Figure 5:**
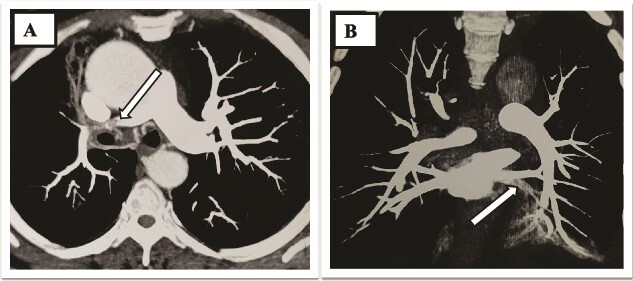
(A and B) Thrombus noted in bilateral lobar pulmonary arteries.

**Figure 6:**
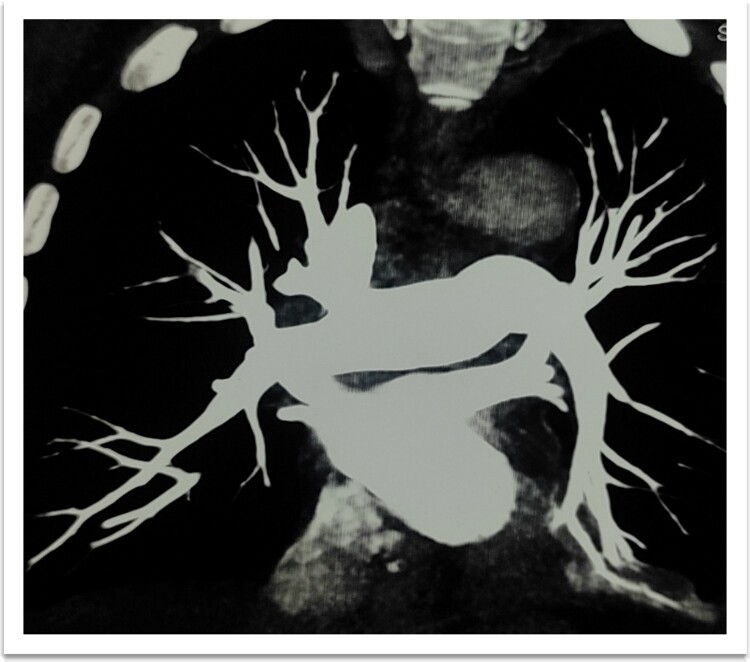
Dilated pulmonary trunk and both main pulmonary arteries.

**Figure 7:**
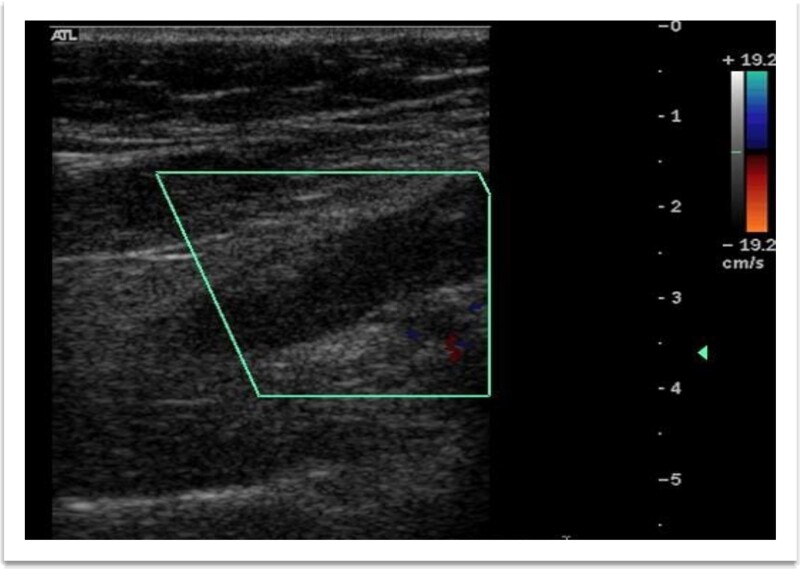
Ultrasonography color Doppler of bilateral lower limb showing complete thrombus in right popliteal vein taking no vascularity.

**Figure 8:**
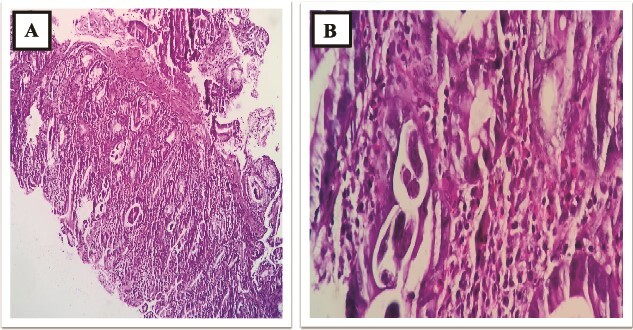
(A and B) D2 biopsy showing infiltration of chronic inflammatory cells (plasma cells, eosinophils and lymphocytes) in lamina propria.

**Figure 9:**
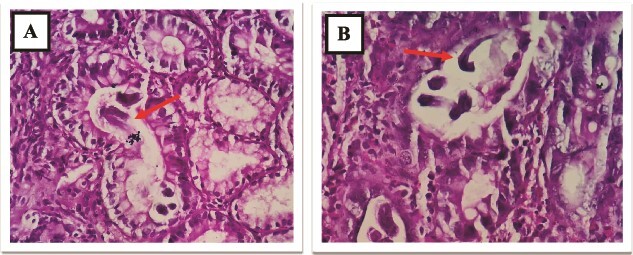
(A and B) D2 biopsy showing larvae of *Strongyloides stercoralis* in duodenal crypts.

**Figure 10:**
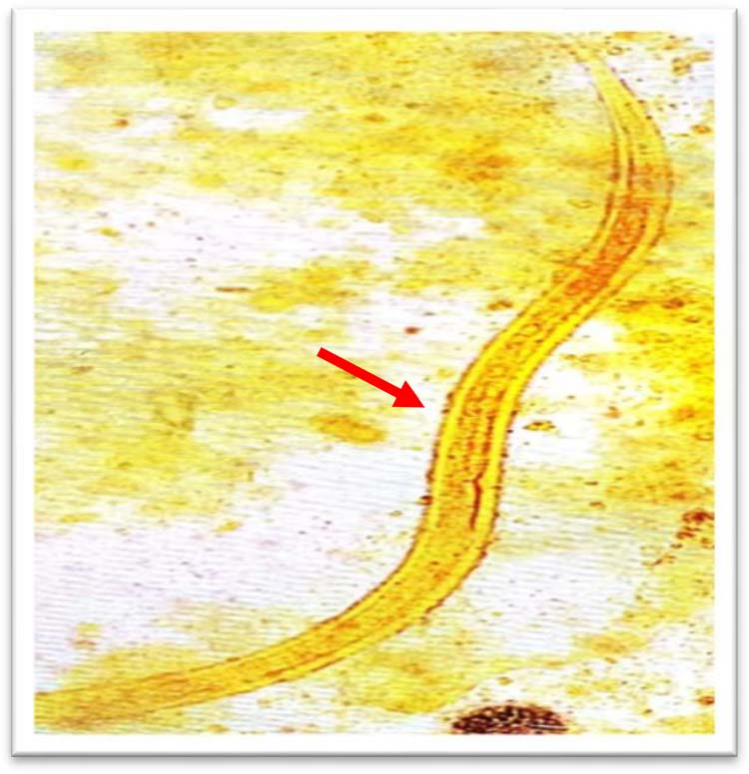
*Strongyloides stercoralis* larva in stool wet mount.

**Figure 11:**
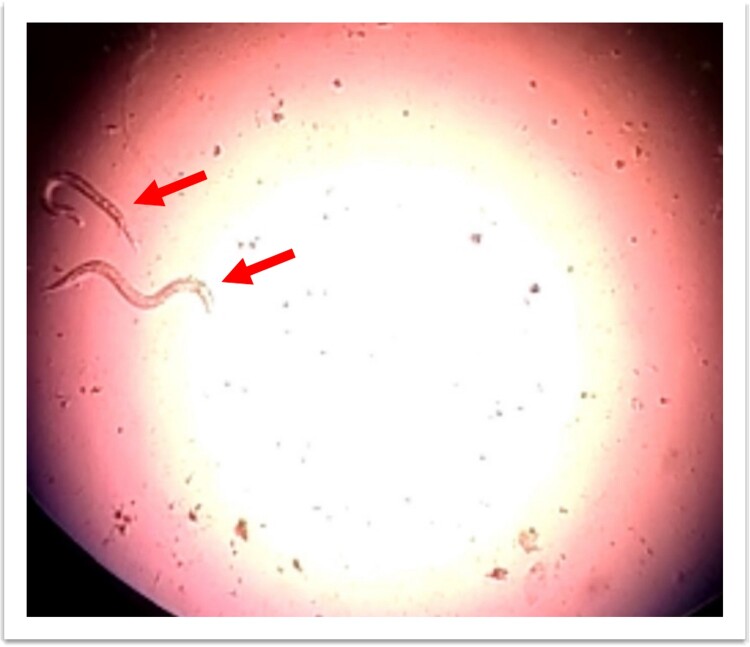
*Strongyloides stercoralis* larva in broncho-alveolar lavage fluid.

A 73-year-old male patient without any comorbidities presented with breathlessness for the last 12 h along with bipedal pitting edema and scrotal edema for the last 1 month with no features suggesting orthopnea or paroxysmal nocturnal dyspnea. There was no history of any medication intake, prolonged steroid abuse, altered bowel habits, recent surgery, or prolonged immobilization. Physical examination revealed tachypnea (respiratory rate—44/min), tachycardia (heart rate—140 beats/min), mild pallor, hypotension (BP 70/40 mmHg) with SpO2 80% in room air and 96% with 8 liters of O2/min via face mask. Peripheral extremities were cold and clammy. Bilateral pitting pedal edema (Fig. 1), and scrotal edema along with leukonychia were present (Fig. 2). Jugular venous pressure was not raised. Cardiovascular, respiratory, gastrointestinal, and lymphoreticular system examinations were within normal limits. For shock, vasopressor (noradrenaline) support and intravenous fluid resuscitation were carried on. The patient survived the episode of shock. 12-lead electrocardiogram showing sinus tachycardia (Fig. 3) and negative cardiac biomarkers ruled out acute coronary syndrome. Chest X-ray was performed which was normal (Fig. 4). 8 a.m. serum cortisol was found to be normal (11.2 mcg/dl). On evaluation, the patient was found to have iron deficiency anemia (transferrin saturation 10%) with normal vitamin B12 levels. Fasting blood glucose was 93 mg/dl with a Glycated hemoglobin (HbA1C) of 5.4. Serology for Human Immunodeficiency virus (HIV)- 1,2; Hepatitis C virus (HCV); and Hepatitis B virus (HBV) was non-reactive. The Mantoux test was negative. Hypoproteinemia (4.4 mg/dl) along with hypoalbuminemia (2.4 mg/dl) was evident with normal renal function test. Markers of inflammation were elevated [erythrocyte sedimentation rate (ESR)-64, Ferritin-732 mcg/L, C-reactive protein (CRP)-12.6 mg/dl]. 24 h of urinary protein was 119 mg/24-h urine. No dyslipidemia was present. Ultrasonography of the whole abdomen revealed no evidence of chronic liver disease or any kidney disease. 2D echocardiography revealed an ejection fraction of 60% with right ventricular free wall hypokinesia without any pericardial effusion. Raising the suspicion of pulmonary thromboembolism, computed tomography of pulmonary angiography was performed where pulmonary thromboembolism was noted in bilateral lower lobe pulmonary arterial branches and right-sided upper lobe pulmonary arterial branches (Fig. 5A and B) with dilated pulmonary trunk and both main pulmonary artery (MPA) [MPA 29 mm, right-22 mm, left-18 mm] (Fig. 6). During the hospital stay, the patient was gradually developing asymmetrical right leg swelling for which an ultrasonography color doppler of bilateral lower limbs was performed where a long segment thrombus was noted extending from the popliteal vein to complete involvement of the external iliac vein (Fig. 7). Antiphospholipid syndrome was ruled out. Protein, C, S, antithrombin III, and Serum homocysteine were within normal limits. Factor V mutation was not detected. Paroxysmal Nocturnal Hemoglobinuria (PNH) profile was negative. No Monoclonal protein band was detected on serum protein electrophoresis (SPEP). A computed tomography scan of the neck, thorax, and abdomen revealed no evidence of internal solid organ malignancy. Anticoagulation was started with low molecular weight Heparin (LMWH) and Warfarin and INR were monitored. To unveil the etiology of iron deficiency anemia and hypoproteinemia, stool examination, and upper gastrointestinal endoscopy were planned which revealed erythematous duodenal mucosa. D2 biopsy was taken which revealed chronic inflammatory cell infiltration in lamina propria consisting of eosinophils, plasma cells, and lymphocytes (Fig. 8A and B) along with the presence of a parasite within the lumen of duodenal crypts morphologically resembling *Strongyloides stercoralis* (Fig. 9A and B). The stool was positive for occult blood and *Strongyloides stercoralis* larva was seen on wet mount (Fig. 10). Bronchoscopy was done where the larval stage of the parasite was seen (Fig. 11). Ivermectin (200 μg/kg orally) was started with Albendazole (400 mg orally two times a day). On continuation of the treatment with anticoagulant and anthelmintic, target INR was achieved (INR 2.2). The patient’s general well-being and appetite were improved within 2 days of therapy.

## Discussion

Strongyloidiasis is a parasitic disease predominantly confined to developing countries of the tropics and subtropics caused by the female nematode *Strongyloides stercoralis*. It is commonly known as “Thread worm” [4]. Being a nematode of the small intestine, the journey of *Strongyloides* begins from the penetration of the skin of its definitive host, the human. The filariform larva, a free-living resident of soil, locates its host via chemicals of skin majority being Urocanic acid [5]. After skin penetration, it enters the bloodstream which carries it up to the lungs where it is swallowed. In the duodenum and jejunum, the larva molts twice to become an adult female. It then lays eggs, excreted in feces, and ultimately hatches to a rhabditiform larva. Autoinfection is a predominant property of this larva, causing parasitemia without any evident clinical signs and symptoms, replicating the dormant nature of the worm. Mostly the symptoms are limited to the intestinal, pulmonary, and integumentary system ranging from mild pain abdomen, loose stools, altered bowel habits, chronic cough, wheezing, and pruritus [6]. Repeated autoinfection causes a significant increase in the burden of the parasite leading to hyperinfection syndrome. In immunocompromised patients, dissemination of the worm to tissues outside the traditional life cycle sites in the human body causes disseminated strongyloidiasis. Increased larval load and deposition of the nematode in various tissues activate the proinflammatory and prothrombotic factors leading to thromboembolic events. *Strongyloides* hyperinfection leads to protein-losing enteropathy, which is also a risk factor for venous thromboembolism [3, 7]. Being a resident in the humid climate of a village in Southeast Asia, with a history of walking barefoot in the fields several times as part of his occupation as a farmer, as in this case, the patient is supposed to have chronic infection with the parasite. This leads to repeated autoinfection and hyperinfection with the parasite thus leading to pulmonary thromboembolism and progressive right lower limb deep vein thrombosis as a complication of the disease. Strongyloides larva identification in stool samples, tissue histopathology, or bronchoalveolar lavage remains the gold standard for the diagnosis of Strongyloides hyperinfection. Luciferase immunoprecipitation system (LIPS) provides the highest positive predictive value with a specificity of 100% but is seldom available in endemic areas as a diagnostic test [8]. RT-PCR can also be done on body fluids and tissue samples but lacks sensitivity. Apart from the management of complications, the primary disease can be easily cured using Ivermectin in association with Albendazole, which are very effective as well as cheap drugs useful for eliminating the parasite [9]. This patient was treated with anticoagulation in view of DVT and pulmonary embolism. Embolectomy may be considered in case of pulmonary thromboembolism after the patient gets hemodynamically stabilized.

## Conclusion

Due to the wide array of clinical manifestations of strongyloidiasis hyperinfection and dissemination, clinicians must maintain a high index of suspicion for rare presentations of this tropical infection, like pulmonary thromboembolism.
